# Genome Sequencing Identified a SARS-CoV-2 Lineage B.1.1.7 Strain with a High Number of Mutations from Dhaka, Bangladesh

**DOI:** 10.1128/MRA.00345-21

**Published:** 2021-05-27

**Authors:** Razib Mazumder, Ahmed Abdullah, Mohammad Enayet Hossain, M. Mahfuzur Rahman, Omar Hamza Bin Manjur, Mustafizur Rahman, Dinesh Mondal

**Affiliations:** a Genomics Center, Laboratory Sciences and Services Division, International Centre for Diarrhoeal Disease Research, Bangladesh (icddr,b), Dhaka, Bangladesh; b Virology Laboratory, International Centre for Diarrhoeal Disease Research, Bangladesh (icddr,b), Dhaka, Bangladesh; Queens College CUNY

## Abstract

We report a coding-complete genome sequence of the severe acute respiratory syndrome coronavirus 2 (SARS-CoV-2) strain SARS-CoV-2/BGD/GC001, isolated from a Bangladeshi patient with respiratory symptoms. Phylogenetic analysis assigned this strain to lineage B.1.1.7, which presented a total of 36 mutations in the spike and other genomic regions compared to strain Wuhan Hu-1 (GenBank accession number NC_045512.2).

## ANNOUNCEMENT

Severe acute respiratory syndrome coronavirus 2 (SARS-CoV-2) belongs to the family *Coronaviridae* and genus *Betacoronavirus* (1). Lineage B.1.1.7, which emerged in the United Kingdom, has attracted particular attention due to its high transmissibility and immune escape potential (2). Herein, we announce a coding-complete genome sequence of the strain SARS-CoV-2/BGD/GC001 (GC001) belonging to lineage B.1.1.7 and detected in a patient with respiratory symptoms who presented on 31 January 2021 in Dhaka, Bangladesh. Ethical approval was obtained from the icddr,b Research and Ethical Review Committee (protocol number PR-21040).

**FIG 1:**
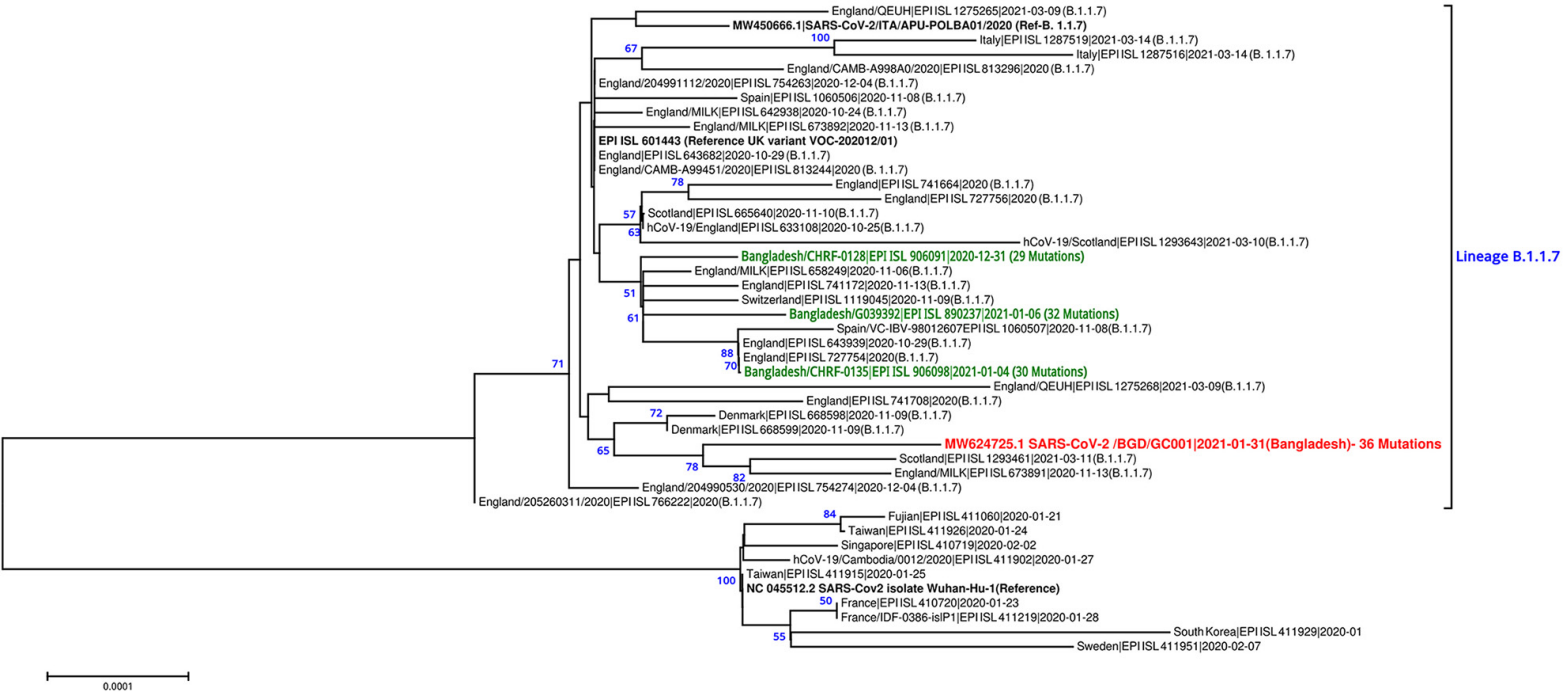
Phylogenetic tree showing the genome sequence of SARS-CoV-2/BGD/GC001/2021 (GenBank accession number MW624725.1) together with 44 sequences, including 34 lineage B.1.1.7 sequences and 10 non-B.1.1.7 lineage sequences retrieved from GISAID and NCBI GenBank. The sequence reported in this announcement is highlighted in red; other B.1.1.7 lineage sequences from Bangladesh are shown in green. The percent bootstrap support values are indicated at each node (values of <50 are omitted).

The specimen (naso-oropharyngeal swabs) from the symptomatic patient was processed for nucleic acid isolation utilizing a QIAamp viral RNA minikit (Qiagen, Germany). SARS-CoV-2 was confirmed using a TaqMan real-time PCR (RT-PCR) assay (3). A cDNA library was prepared utilizing the Illumina TruSeq stranded total RNA Gold low-throughput (LT) library preparation kit following the manufacturer’s instructions; rRNA reduction was performed using the Ribo-Zero Gold protocol. The libraries were normalized to 10 nM following Illumina’s standard normalization method and pooled using 10 μl of each library, which was then sequenced using the NextSeq v2.5 midoutput kit (2 × 150 cycles) on the NextSeq 500 instrument at the Genomics Center of the icddr,b in Dhaka, Bangladesh. There were 112,690,904 paired-end raw reads, whose quality was assessed using FastQC v0.11.11 (4). The adapters were trimmed using Trimmomatic v0.39 based on Q30 values with the following parameters: window size, 4; Phred quality, 15; and minimum read length, 40 (5). After trimming, 205,697,670 reads were used for reference-based alignment. The SARS-CoV-2-specific reads were mapped and filtered using SMALT v0.7.6 (http://www.sanger.ac.uk/science/tools/smalt-0) and SAMtools v1.9 (6). *De novo* assembly was performed with 17,324 reads using SPAdes v3.11.1 (7), and the quality was assessed using QUAST v5.0.2 (8). RATT was employed to annotate the genome with Wuhan-Hu-1 as the reference strain (GenBank accession number NC_045512.2) (9). Mutations were assessed utilizing the Genome Detective Coronavirus Typing Tool (10). The Nextstrain and PANGOLIN Web tools were used for comparative genomics (11, 12). MUSCLE v3.8.31 (13) and MEGA7 (14) software were used to generate a phylogenetic tree using the neighbor-joining method with 1,000 bootstraps (Fig. 1). Default parameters were applied for all tools unless otherwise mentioned.

The genome sequence of strain GC001 was 29,842 bp long with a G+C content of 37.98%. Phylogenetic analysis assigned strain GC001 to lineage B.1.1.7, which is a predominant lineage worldwide (Fig. 1). We identified 33 mutations and 3 deletions in GC001 in comparison to strain Wuhan-Hu-1 (GenBank accession number NC_045512.2) (Table 1). Strain GC001 showed a high number of mutations compared to other SARS-CoV-2 lineage B.1.1.7 strains identified in Bangladesh at the time of the analysis (Fig. 1). Moreover, strain GC001 did not cluster closely with other B.1.1.7 genomes identified from Bangladesh to date, which hints toward its independent introduction into the country. These observations suggest the importance of genome sequencing of SARS-CoV-2 samples from travelers, particularly those returning from high-risk countries.

**TABLE 1:** Mutations of SARS-CoV-2 strain GC001 (GenBank accession number MW624725.1) in comparison to the reference strain (NC_045512.2)

Gene or region	Mutation no.	CDS codon position^*a*^	Amino acid change	Nucleotide position	Nucleotide change
5′ untranslated region	1			241	C > T
ORF1ab	2	216		913	C > T
	3	615		2110	C > T
	4	924		3037	C > T
	5	1001	T > I	3267	C >T
	6	1708	A > D	5388	C > A
	7	1907		5986	C > T
	8	2230	I > T	6954	T > C
	9	2573		7984	T > C
	10	3198		9857	C > T
	11	3675–3677	SGF deletion	11288–11296	Deletion TCTGGTTTT
	12	4619	P > L	14120	C > T
	13	4715	P > L	14408	C > T
	14	4804		14676	C > T
	15	505		15279	C > T
	16	5304		16176	T > C
	17	6376	P > S	19390	C > T
S	18	69–70	HV deletion	21766–21771	Deletion ACATGT
	19	75	G > V	21786	G > T
	20	144	Y deletion	21992–21994	Deletion TAT
	21	501	N > Y	23063	A > T
	22	570	A > D	23271	C > A
	23	614	D > G	23403	A > G
	24	681	P > H	23604	C > A
	25	716	T > I	23709	C > T
	26	982	S > A	24506	T > G
	27	1118	D > H	24914	G > C
ORF8	28	27	Q > stop	27972	C > T
	29	52	R > I	28048	G > T
	30	68	K > stop	28095	A > T
	31	73	Y > C	28111	A > G
N	32	3	D > L	28280–28282	GAT > CTA
	33	203	R > K	28881–28882	GG > AA
	34	204	G > R	28883	G > C
	35	235	S > F	28977	C > T
	36	269		29080	T > C

a CDS, coding DNA sequence.

### Data availability.

The genome sequence of SARS-CoV-2/BGD/GC001 was deposited in the NCBI database under the BioProject accession number PRJNA702998, BioSample accession number SAMN17993365, and GenBank accession number MW624725.1. The Illumina raw reads have been deposited in the NCBI Sequence Read Archive under accession number SRR13744683.
